# The association between urbanization and child height: a multilevel study in China

**DOI:** 10.1186/s12889-019-6921-z

**Published:** 2019-05-14

**Authors:** Yan Zhang, Han Wang, Xi Wang, Meicen Liu, Yinping Wang, Yan Wang, Hong Zhou

**Affiliations:** 1 Department of Women’s Health, Beijing Obstetrics and Gynecology Hospital, Capital Medical University, Beijing Maternal and Child Health Care Hospital, No.251 Yaojiayuan Road, Chaoyang District, Beijing, 100026 China; 2 Department of Maternal and Child Health, School of Public Health, Peking University, No.38 Xueyuan Road, Haidian District, Beijing, 100191 China; 3 The Children’s Hospital of Philadelphia, 2716 South Street, Philadelphia, PA 19146 USA

**Keywords:** Child height, Multilevel study, Urbanization index, Community factors

## Abstract

**Background:**

Recent economic development in China has been accompanied by well-documented health inequalities between regions. The impact of individual factors on child height has been widely studied, but the influence of community-level factors has not yet been fully studied.

**Methods:**

The cross-sectional data of 1606 Chinese children aged 5–18 years from the China Health and Nutrition Survey 2011 were used. Multilevel analysis was used to examine the association of community factors (using urbanization index) with child height. Child height was measured following standardized procedures, and height-for-age Z scores were calculated as outcome variables. Datasets were presented at two levels: community variable (Level-2) was an urbanization index which is a composite score summarizing 12 community-level contextual factors. Individual variables (Level-1) were child gender, ethnicity, percentage of energy intake from protein, maternal height, maternal education level, and family income.

**Results:**

Urbanization index was associated with child height. Among the 12 community-level factors, ‘education’ were positively associated with child height. Additionally, stratified analyses by age showed that ‘population density’ and ‘housing’ were positively significantly associated with the height of elder children (13–18 years). At the individual level, male sex, higher maternal height, higher maternal education levels, higher family income, and higher percentage of energy intake from protein, were significantly positively associated with child height.

**Conclusions:**

Our findings point to the role of contextual factors that generate differences between regions in shaping the distribution of child physical health outcomes. Our study suggests that public health programs and policies for child’s physical development may need to combine individual-centered strategies and also approaches aimed at changing residential environments.

## Background

The growth and development of children is directly related to their current and future health status. The height of children is an important indicator of growth and development [1]. Height is a multifactorial characteristic which is affected by genetic and environmental factors [2]. Early childhood low height-for-age is associated with a series of irreversible adverse outcomes, including athletic development retardation, cognitive impairment, and poor academic performance [3]. Therefore, it is important to identify the environmental factors associated with height so as to improve the overall health status of children.

The average height of Chinese children has increased in the past two decades [4], possibly related to the improvement of individual factors, such as protein intake [5], family income [6, 7] and maternal education level [8, 9]. These improvements of individual factors resulted from China’s economic and social development through increased food supply, better housing conditions, and improved health care services [10].

Chinese government has made great efforts to fill the gap of maternal and child health inequalities between urban and rural areas by explicit policy of ensuring safe birth in health facilities, huge investment in human resources to improve the number of licensed doctors, reducing financial barriers and providing childbirth subsidies, and many other public health programs [11]. However, the rural/urban disparities that concerning health services still exist [12, 13]. A study [14] evaluated the effects of China’s economic reforms on the growth of children, and showed an increase in the average height of children in both rural and urban areas, while this increase in urban areas was five times that in rural areas. In developing counties like China, urbanization has been found to improve child health outcomes such as lower under-five mortality and lower undernutrition rate [15, 16], through increasing access to health care services, education resources, and safe water supply [17]. Smith et al. [18] also indicated that in developing counties urban children were more likely to have well-nourished mothers, which meant better prenatal and birthing care, better feeding practices and greater use of health services for preventive and curative care, result in improved growth and lower morbidity. However, whether and how urbanization is associated with children’s physical development in China remain unclear. Understanding the relationship between community-level contextual factors can help explaining the disparity in children’s physical development between regions and inform the approaches for improving child’s health.

In this study, we investigated the effects of urbanization index (a composite score summarizing 12 community-level contextual factors) on child height among Chinese children aged 5–18 years old from the China Health and Nutrition Survey (CHNS) in 2011.

## Methods

### Study population

This study used secondary data from the 2011 dataset of the CHNS, which was a large survey conducted in nine provinces (Heilongjiang, Liaoning, Jiangsu, Shandong, Henan, Hubei, Hunan, Guangxi and Guizhou) and three mega-cities (Beijing, Shanghai and Chongqing) in China. A multistage, stratified sampling design was conducted to ensure that CHNS provided a fair representation of urban and rural areas. Using sampling strategy, two cities and four counties per province were selected based on income (low, middle and high). Within cities, two urban and two suburban communities were randomly selected based on urbanicity. Within counties, one community in the capital city and three rural villages were randomly chosen, also based on urbanicity. Twenty households per community were then randomly selected for participation. Survey protocols, instruments, and the process of obtaining informed consent for this study were approved by the institutional review committees of the University of North Carolina at Chapel Hill as well as the National Institute for Nutrition and Health, which is affiliated with the China Center for Disease Control and Prevention. Participants provided written, informed consent. Data, documentation, and details on sampling, representativeness, and validity of data can be accessed through the Carolina Population Center website: (http://www.cpc.unc.edu/projects/china).

A total of 1712 children between 5 and 18 years old from 290 communities was enrolled in the 2011 dataset. We excluded participants with major medical conditions (1 child with diabetes, 1 child with wheezing, and 22 children with bone fractures), and had missing information on main variables (77 children). Finally our study included 1606 observations in 272 communities.

### Outcome variable

#### Physical height of child

Height was measured without shoes using a portable stadiometer and recorded to the nearest tenth of a centimeter. The height for age Z score (HAZ), which is an indicator standardized for age and sex, was calculated to evaluate the height levels of children and adolescents. Higher HAZ indicates better height growth. WHO Growth Reference 2007 was used. This reference is a reconstruction of the 1977 National Center for Health Statistics (NCHS)/ WHO reference, and it is recommended by WHO as a growth reference for children and adolescents aged 5–19 years [19]. SAS macro was used for calculation as WHO recommended (http://www.who.int/childgrowth/software/en/).

### Community level variable

#### Urbanization index

Urbanization level of each community/village was measured using urbanization index. The urbanization index was developed by Jones-Smith and Popkin [20] using in the CHNS. It was defined as a 12-component index, capturing community-level physical, social, cultural, and economic environments designed and validated for the CHNS. Urbanicity Scale is used for measurement of the 12 component (Table 1). Each component was given a score from 0 to 10, and then weighted equally in the overall index and added together for an overall maximum possible score of 120. Higher scores indicate greater urbanization. This scale has been validated for content validity, reliability (ɑ = 0.85–0.89), and stability (r = 0.90–0.94) [20]. We represented urbanization by quintiles of an urbanization index reflecting population size and density and community infrastructure.

**Table 1:** Description of urbanization component index

urbanization components	Description
Communication	Media availability in the community and percent of households with electronics
Population Density	Population per km^2^
Diversity	Community variance in education and income levels
Economic Activity	Daily wage for average male worker and % community engaged in nonagricultural labor
Health Infrastructure	Type, distance and number of health services (clinics, hospitals, pharmacies, etc.) in the community
Housing	Availability of electricity, water, gas
Traditional Markets	Types, distances and business hours of food and fuel markets
Social Services	Availability of insurance and child care centers
Transportation	Road types and availability of transit services
Education	Average highest attained level for adults
Modern Markets	Quantity of supermarkets and modern eating establishments
Sanitation	Availability of treated water and presence of excrement in public space

### Individual level variables

#### Maternal education

CHNS classified maternal education level as follows: no school (0 year), primary school (1–6 years), junior middle school (1–3 years), senior middle school (1–3 years), middle technical or vocational school (1–2 years), college (3–4 years in college/university), and graduate school (over 4 years in college/university). Maternal education level was then categorized into 3 groups: low education(≤6 years of education); middle education (7 to 12 years of education); high education(≥12 years of education).

#### Family income

In CHNS, household income was evaluated by household income per capita inflated to 2011. With the equalized household income values, we created four break points to define income quintiles (1st to 5th quintile), with the 1st quintile set as the baseline.

#### Percentage of energy intake from protein

Dietary intake was assessed using three consecutive in-person 24-h recalls randomly allocated to begin between Monday and Sunday, in combination with a household food inventory conducted over the same 3-day period. Dietary data in children was based on a parent-assisted self-report (child age < 10 years) or self-report (child age ≥ 10 years) questionnaire. Food models and picture aids were used by trained interviewers to assist with estimating portion sizes. For dishes prepared at home, recipe components were taken from the household food inventory, and portion sizes were based on the proportion of the dish reportedly consumed by the participant. Energy and nutrient intake levels for dietary data were calculated using the China Food Composition Table. Details of the process can be accessed through the Carolina Population Center website: (http://www.cpc.unc.edu/projects/china).

### Statistical analysis

Descriptive statistics on individual demographic variables were calculated. Continuous variables were presented as means and standard deviations (SD). Categorical variables were expressed as percentages. The present study focused on cross-sectional analysis of CHNS data in 2011. We applied multilevel models and statistical software SPSS 21.0 to conduct a two-level regression analysis. We examined the relationship between urbanization index and child height after controlling for individual variables. The modelling was done in four steps: Model 1 (empty model) examined only within-group homogeneity. Model 2 included the community-level contextual variable (urbanization index). In model 3, the individual-level explanatory variables were added to Model 2. These individual variables consisted of child gender, age, maternal education level, maternal height, family income per capita, and percentage of energy intake from protein. Then in Model 4, the urbanization index was modified to 12 components of the urbanization index. The individual variables were the same as in Model 3. Finally, subgroup analysis was conducted to explore the heterogeneous effects by different age groups (5–12 years and 13–18 years). In each model, the inter-class correlation (ICC) was calculated as the ratio of between-community variance to total variance in children’s heights.

## Results

Demographic and anthropometric measurements are shown in Table 2. In total, 1606 children (827 boys and 779 girls, mean age 11.14 ± 3.71 years) were included. Among them, 91.47% were Han ethnicity. The average percentage of energy intake from protein was (0.14 ± 0.03%). The average height-for-age Z score was − 0.19 ± 1.24. The proportion of height-for-age Z score below 1 was 85.24%. For the maternal education level, the percentage of ‘no school, primary school and middle school’ was 80.45%. Both family income per capita and urbanization index were categorized by quintiles (Table 2).

**Table 2:** Descriptive statistics of individual level variables among children aged 5–18 years from the China Health and Nutrition Surveys, 2011

Variable	N	Percentage (%) or x ± s
gender		
Male	827	51.49
Female	779	48.51
Age (year)	1606	11.14 ± 3.71
Ethnicity		
Miao	48	2.99
Buyi	52	3.24
Tujia	37	2.30
Han	1469	91.47
Family income per capita		
1st Quintile	336	20.92
2nd Quintile	310	19.30
3rd Quintile	320	19.93
4th Quintile	312	19.43
5th Quintile	328	20.42
Maternal education level		
low education	573	35.68
middle education	829	51.62
high education	204	12.7
Maternal height	1289	
Percentage of energy take from protein (%)	1606	0.14 ± 0.03
Urbanization Index		157.71 ± 6.10
1st quintile	314	19.55
2nd quintile	319	19.86
3rd quintile	318	19.80
4th quintile	342	21.30
5th quintile	313	19.49
Z score of height for age	1606	−0.19 ± 1.24
< −2	111	6.91
−2~	265	16.50
−1~	514	32.00
0~	479	29.83
1~	188	11.71
> 2	49	3.05

Table 3 shows the results of multilevel models. Model 1 constructed an empty model, because the intercept of random effect was statistically significant (*P* < 0.01), The inter-class correlation (ICC) coefficient in Model 1 was 0.26, suggesting between-community heterogeneity in children’s height. Model 2 shows the fixed effects results for community-level factors. Urbanization was significantly associated with an increase of height-for-age Z score. Compared with 1st quintile, children living in a community with higher urbanization levels (3rd, 4th and 5th quintile) reported higher height-for-age Z scores (*p* < 0.05). Model 3 added individual and community variables. Boys, higher maternal height, higher maternal education levels, higher family income and higher percentage of energy intake from protein were significantly positively associated with height-for-age Z score. Elder age was significantly negatively associated (p < 0.05). After adjustment for individual predictors, urbanization index remained significantly positively associated with height-for-age Z score. Compared with the 1st quintile, children from a community with higher urbanization levels (3rd, 4th and 5th quintile) were more likely to report better height-for-age Z scores. After adjustment for individual predictors, Model 4 showed that ‘education’ was significantly associated with height-for-age Z score. Other urbanization components were not significantly associated. The results of individual variables were consistent with Model 3 except for maternal education level.

**Table 3:** Coefficients from two-level linear regression models for height among children aged 5–18 years, 2011

effects	Model 1			Model 2			Model 3			Model 4		
	Coefficient	SE	P	Coefficient	SE	P	Coefficient	SE	P	Coefficient	SE	P
*Fixed effects*												
Intercept	−0.10	0.05	0.04	− 0.59	0.10	< 0.01	−9.76	0.86	< 0.01	−9.69	0.89	< 0.01
Individual variables- Level-1												
Age (year)							−0.05	0.01	< 0.01	− 0.05	0.01	< 0.01
Gender (ref. = female)							0.13	0.06	0.04	0.13	0.06	0.03
Ethnicity (ref. = Han)												
Miao							−0.10	0.22	0.67	−0.13	0.22	0.55
Buyi							−0.34	0.23	0.17	−0.20	0.23	0.40
Tujia							−0.37	0.28	0.20	−0.37	0.27	0.20
Maternal height							0.06	0.01	< 0.01	0.06	0.01	< 0.01
Maternal Education level (ref. = low school)												
middle school							0.10	0.08	0.22	0.05	0.08	0.53
high school							0.39	0.12	< 0.01	0.18	0.13	0.15
Family income per capita (ref. = 1st Q)												
2nd Quintile							0.19	0.10	0.07	0.18	0.10	0.07
3rd Quintile							0.26	0.10	0.01	0.25	0.10	0.01
4th Quintile							0.23	0.10	0.03	0.22	0.10	0.03
5th Quintile							0.30	0.11	0.01	0.25	0.11	0.02
Energy intake from protein (%)							3.01	0.96	< 0.01	2.40	0.97	0.01
*Community variables- Level-2*												
Urbanization Index (ref. = 1st Q)												
2nd Quintile				0.19	0.14	0.18	0.14	0.13	0.28			
3rd Quintile				0.62	0.14	< 0.01	0.26	0.13	0.04			
4th Quintile				0.75	0.14	< 0.01	0.25	0.13	0.06			
5th Quintile				0.82	0.14	< 0.01	0.39	0.14	< 0.01			
Urbanization components												
Communication										0.02	0.04	0.69
Population Density										0.05	0.03	0.10
Diversity										0.02	0.04	0.62
Economic Activity										0.01	0.02	0.56
Health Infrastructure										0.01	0.02	0.87
Housing										−0.05	0.04	0.22
Traditional Markets										− 0.01	0.01	0.15
Social Services										−0.02	0.01	0.15
Transportation										−0.01	0.02	0.77
Education										0.13	0.03	< 0.01
Modern Markets										−0.03	0.02	0.17
Sanitation										0.02	0.02	0.29
*Random effects*												
Intercept	0.40	0.05	< 0.01	0.29	0.04	< 0.01	0.12	0.03	< 0.01	0.10	0.03	< 0.01
Residual	1.11	0.04	< 0.01	1.12	0.04	< 0.01	1.01	0.04	< 0.01	1.00	0.04	< 0.01
-2LL	5009.10			4963.70			3653.30			3625.50		
ICC	0.26			0.20			0.21			0.09		

Model 1: empty model; Model 2: added urbanization index in community level from Model 1; Model 3: added individual and community variables from Model 2; Model 4: added urbanization components instead of urbanization index in community level from Model 3. The inter-class correlation coefficient (ICC) is a ratio of between-community variance to total variance in children’s heights.-2LL: negative twice likelihood ratio.

Subgroup analysis was further conducted according to different age groups (5–12 year, 13–18 years). Results were shown in Table 4. In individual level, energy intake from protein was positively associated with height-for-age Z score only in 5–12 year group (*p* < 0.05), and child gender was no longer significantly associated in either subgroup. In community level, only one urbanization component ‘education’ was positively associated with height for age Z score in 5–12 year group(*p* < 0.05), while three urbanization components ‘population density’, ‘housing’ and ‘education’ were positively associated with height for age Z score in 13–18 year group (*p* < 0.05).

**Table 4:** Coefficients from two-level linear regression models for height among children, stratified by child’s age, 2011

effects	5–12 years						13–18 years					
	Model 5			Model 6			Model 7			Model 8		
	Coefficient	SE	P	Coefficient	SE	P	Coefficient	SE	P	Coefficient	SE	P
*Fixed effects*												
Intercept	−9.18	1.12	< 0.01	−9.26	1.17	< 0.01	−13.19	1.23	< 0.01	−12.23	1.27	< 0.01
Individual variables- Level-1												
Gender (ref. = female)	0.13	0.08	0.10	0.14	0.08	0.09	0.08	0.08	0.34	0.10	0.08	0.24
Ethnicity (ref. = Han)												
Miao	−0.09	0.27	0.76	− 0.11	0.27	0.69	−0.30	0.36	0.56	−0.29	0.34	0.55
Buyi	−0.41	0.33	0.26	−0.28	0.34	0.43	−0.20	0.26	0.58	−0.05	0.25	0.87
Tujia	−0.57	0.35	0.15	−0.53	0.35	0.18	−0.13	0.36	0.78	−0.10	0.34	0.82
Maternal height	0.05	0.01	< 0.01	0.05	0.01	< 0.01	0.08	0.01	< 0.01	0.08	0.01	< 0.01
Maternal Education level (ref. = low school)												
middle school	0.12	0.10	0.24	0.09	0.10	0.38	0.06	0.11	0.58	−0.06	0.11	0.57
high school	0.55	0.16	< 0.01	0.37	0.17	0.03	0.27	0.17	0.11	−0.07	0.18	0.68
Family income per capita (ref. = 1st Q)												
2nd Quintile	0.18	0.13	0.18	0.19	0.13	0.14	0.23	0.16	0.15	0.18	0.15	0.23
3rd Quintile	0.35	0.13	< 0.01	0.35	0.13	0.01	0.14	0.15	0.36	0.10	0.15	0.50
4th Quintile	0.31	0.13	0.02	0.30	0.14	0.03	0.11	0.15	0.47	0.07	0.15	0.63
5th Quintile	0.29	0.14	0.04	0.24	0.14	0.09	0.31	0.15	0.04	0.21	0.15	0.16
Energy intake from protein (%)	3.44	1.25	< 0.01	2.83	1.26	0.03	1.65	1.40	0.24	1.22	1.36	0.37
*Community variables- Level-2*												
Urbanization Index (ref. = 1st Q)												
2nd Quintile	0.10	0.16	0.51				0.14	0.18	0.42			
3rd Quintile	0.24	0.16	0.14				0.19	0.17	0.25			
4th Quintile	0.21	0.17	0.20				0.22	0.18	0.21			
5th Quintile	0.36	0.17	0.04				0.35	0.18	0.05			
Urbanization components												
Communication				0.03	0.05	0.50				−0.02	0.04	0.67
Population Density				0.05	0.04	0.23				0.09	0.04	0.01
Diversity				−0.01	0.05	0.86				0.08	0.05	0.09
Economic Activity				0.02	0.02	0.49				−0.01	0.02	0.83
Health Infrastructure				< 0.01	0.02	0.88				< 0.01	0.02	0.85
Housing				−0.03	0.05	0.54				− 0.11	0.05	0.03
Traditional Markets				−0.01	0.02	0.55				−0.01	0.02	0.57
Social Services				− 0.02	0.02	0.32				−0.02	0.02	0.15
Transportation				−0.02	0.03	0.38				0.04	0.03	0.08
Education				0.11	0.04	0.01				0.15	0.04	< 0.01
Modern Markets				−0.03	0.03	0.22				−0.01	0.03	0.67
Sanitation				0.03	0.03	0.31				0.02	0.03	0.57
*Random effects*												
Intercept	0.16	0.05	< 0.01	0.15	0.05	< 0.01	0.06	0.04	0.06	0.01	0.03	0.40
Residual	1.15	0.07	< 0.01	1.13	0.06	< 0.01	0.70	0.06	< 0.01	0.70	0.06	< 0.01
-2LL	2491.5			2477.2			1118.9			1083.8		
ICC	0.12			0.12			0.08			0.01		

Table 4 presents two-level linear regression analyses stratified by children’s age: Models 5 and 6 were fitted in the age stratum of 5–12 years old, and Models 7 and 8 were fitted in the age stratum of 13–18 years old. For exploratory variables, Models 5 and 7 included the composite urbanization index as community-level contextual factor, while Models 6 and 8 included the 12 urbanization components as community-level variables; all the models have adjusted individual-level factors. The inter-class correlation coefficient (ICC) is a ratio of between-community variance to total variance in children’s heights.-2LL: negative twice likelihood ratio.

## Discussion

In the current study, we found that urbanization index was associated with child height. Among the 12 components of urbanization index, ‘education’ was associated with child height in multilevel models controlling for clustering by community. Additionally, ‘population density’ and ‘housing’ were associated with height of adolescents aged 13–18 years.

Generally, our results were consistent with previous studies. Smith et al. [18] reported that urban children generally have better nutritional status than rural children in developing countries. Eckert et al. [21] summarized that urbanization was associated with a lower risk of undernutrition of children. In China, with the rapid economic growth, the inequalities between urban and rural areas was obvious. The effect of urbanization on child health reflected the inequality between urban and rural areas [22].

We observed a significant association between child height and the ‘education’ segment of the urbanization component. Some studies have documented a positive impact of parental education on child height [8, 9]**.** However, less is known about the effect of community mean education relating to child height. As better education levels often correlate with better family income, people with better education level may choose to live in better communities with improved health-related infrastructure, as well as better educational institutions for their child. Those parents with better education level may respond better to community health educational and health promotion efforts, and may pay more attention to their children’s growth and development. Therefore, these children may have healthier lifestyle habits, diet habits, and health care. Therefore, improving a population’s education level, as well as strengthening community health services, may have important practical and theoretical significance.

The influence of urbanization on height seemed different in younger and elder age groups. In the present study, we divided all the participants into two age groups, 5–12 years and 13–18 years. Because most of the Chinese children go to middle-school at the age of 13, their education environment and life styles might be quite different from children under 12 who were commonly in primary school. According to the result, children aged 13–18 years were more likely affected by urbanization, with three statistically significant factors (population density, housing and education). This result was similar to previous studies [23]. Generally, elder children acquire more physical, emotional, cognitive, social and economic resources that have effect on health [24]. But the pathways of population density and housing influencing child height need further study. Higher population density and better housing reflect higher urbanization level, that may help elder children to experience less poverty, better social environment and health services, and lead to a better physical development.

Regarding influence factors at the individual level, gender [25], family income [6, 7], protein intake [5] maternal education [8, 9], and maternal height [26] were previously demonstrated to be associated with child height. These findings were consistent with our findings. Because the present study is a cross-sectional design, these factors may provide important reference points for making health policy at the individual level.

There are some major strengths of this study. First, the urbanization index is a more comprehensive composite index, based on the specific study region (CHNS). It is typically applied to assess the socioeconomic environment of a community in China. Using the urbanization index may produce better findings regarding the relationship between community socioeconomic conditions and child height. Second, in most previous studies, a single-level model was commonly used which ignored the inter-correlation of children within same community. We overcame these limitations by using a multilevel model. A multilevel design can help researchers to examine relevant community-level characteristics that may be associated with child height, and may provide evidence to identify disadvantaged communities and help children living there.

This study also has some limitations. First, as we all know, the CHNS is a longitudinal structure data with several waves, but we only used the data of 2011. The main consideration was that Chinese society has experienced rapid urbanization during the recent decade, by the time we did the data analysis, the newest data was the data in 2011, which reflect the latest condition of Chinese Society. Second, though multi-level model was meaningful for the current data, the sample size of each community was small. Large-scaled study is needed in further research. Third, the study was a cross-sectional design, which cannot explain the causal relationships between child height and urbanization. Further prospective study design was needed. Forth, individual factors which may be associated with child height were limited in the current study, and more individual factors are needed to be involved in further studies, for example genetic factors, excises, etc.

## Conclusion

Community-level contextual factors were significantly associated with child height, especially for elder children, in a representative national population in China. Our findings points to the role of contextual factors that generate differences between regions in shaping the distribution of child physical health outcomes. Our study suggests that public health programs and policies for child’s physical development may need to combine individual-centered strategies and also approaches aimed at changing residential environments.

## Abbreviations

CHNS: China Health and Nutrition Survey
ICC: The inter-class correlation.
SD: Standard deviations
